# A One-Stop Protocol to Assess Myocardial Fibrosis in Frozen and Paraffin Sections

**DOI:** 10.3390/mps5010013

**Published:** 2022-01-27

**Authors:** Divya Sridharan, Nooruddin Pracha, Julie A. Dougherty, Ali Akhtar, Syed Baseeruddin Alvi, Mahmood Khan

**Affiliations:** 1 Department of Emergency Medicine, The Ohio State University, Columbus, OH 43210, USA; Divya.Sridharan@osumc.edu (D.S.); Pracha.1@osu.edu (N.P.); Julie.Dougherty@nationwidechildrens.org (J.A.D.); Akhtar.26@osu.edu (A.A.); Syed.alvi@osumc.edu (S.B.A.); 2 Department of Physiology and Cell Biology, The Ohio State University, Columbus, OH 43210, USA

**Keywords:** histology, Trichrome Staining, cryogenic tissue sections, formalin-fixed paraffin-embedded tissues

## Abstract

Masson’s Trichrome Staining (MTS) is a useful tool for analyzing fibrosis in a plethora of disease pathologies by differential staining of tissue components. It is used to identify collagen fibers in different tissues like heart, lung, skin, and muscles. Especially in cardiac fibrosis, MTS stains the collagen fibers (blue color), which helps in the distinction of scar area versus the healthy area (red color). However, there are several challenges to stain both paraffin-embedded sections and frozen (cryosections) using a single protocol. Therefore, the goal of this study was to develop a simple short protocol to assess cardiac fibrosis in both paraffin-embedded and cryo heart sections. MTS uses three different stains, i.e., Weigert’s Iron Hematoxylin, Biebrich scarlet-acid fuchsin, and aniline blue to detect nuclei, cytoplasm, and collagen, respectively. In this study, we developed a simple short protocol that can be adapted by any lab to easily assess cardiac fibrosis in paraffin and frozen heart sections. Furthermore, we have addressed the challenges that are commonly faced during the immunostaining process and troubleshooting techniques. Overall, we have successfully developed a simple one-step protocol to assess myocardial fibrosis in paraffin-embedded and frozen cryosections.

## 1. Introduction

Myocardial infarction (MI) occurs when an atherosclerotic plaque ruptures in the inner lining of the left anterior descending (LAD) artery leading to the blockage and restriction of blood flow and oxygen supply to the left ventricular heart muscle [1,2]. Nearly a billion cardiomyocytes are lost in a single ischemic cardiac event, which eventually leads to the formation of a non-contractile fibrous scar tissue [2,3]. The size and thickness of the scar tissue provides an estimate of the extent of MI and loss of cardiac function [4]. Hence, analysis of the fibrotic tissue is an important tool in understanding the disease pathology and developing potential therapeutic strategies for MI [5,6]. Collagen type I is upregulated in the scar tissue compared to the healthy heart tissue [7]. This differential expression of collagen can be used to determine the extent of fibrosis in ischemic heart tissues.

Masson’s Trichrome Staining (MTS), first developed by Claude Pierre Masson, has been routinely used to differentiate cells and their components from the surrounding connective tissues. MTS allows for differential color staining of cell nuclei (dark red), collagen (blue), and cell cytoplasm (red/purple) by using an iron hematoxylin dye called Weigert’s Hematoxylin, Aniline Blue, and Biebrich scarlet-acid fuchsin stains, respectively. The tricolor staining of tissues using MTS has been used to detect and quantify fibrosis in numerous tissue types, including liver [8], mammary glands [9], skin [10], bone [11], and heart [12]. This paper describes a detailed methodology to detect and analyze fibrosis in rodent models for MI, using MTS, applicable to both paraffin-embedded and frozen tissue samples.

## 2. Experimental Design

Prepare all reagents using distilled water and analytical grade reagents. Prepare and store all reagents at room temperature (unless mentioned otherwise). Prepare all organic solvents in a chemical fume hood and use the appropriate personal protective equipment as recommended by the SDS.

### 2.1. Tissue Fixation and Embedding

#### 2.1.1. Embedding in Paraffin

1. Phosphate-buffered saline (PBS)
2. 4% Paraformaldehyde (PFA) in PBS
3. Ethanol gradients: 100%, 85%, 70%, 50%, 30%, 10% ethanol in water
4. Xylene
5. Xylene-ethanol solution: Mix equal volumes of ethanol and xylene
6. Paraffin-ethanol mixture: Melt paraffin at 60 °C. Mix equal volumes of melted paraffin and ethanol and place the mixture at 60 °C.
7. Paraffin, melted at 60 °C.
8. Tissue embedding cassettes
9. Tissue embedding molds
10. Heart slicer matrix (2 mm)
11. Blades
12. Forceps
13. Reagent reservoirs
14. Glass beakers
15. Hot plate
16. Heat protective gloves
17. Bucket of ice
18. Water bath
19. Slide warmer

#### 2.1.2. Embedding in Optimal Cutting Temperature (OCT) Compound

1. PBS
2. OCT compound
3. Cryomolds
4. Forceps
5. Dry ice or liquid nitrogen

### 2.2. Tissue Sectioning

1. Superfrost Plus slides (Cat # 12-550-15, Fisherbrand)
2. Microtome (paraffin-embedded samples) or cryostat (frozen samples)

### 2.3. Reagents for MTS

1. MTS Kit (Cat #/HT15-KT Sigma Aldrich, Saint Louis, MO, USA)
2. 5% Phosphomolybdic acid
3. 5% Phosphotungstic acid
4. Biebrich Scarlet-Acid Fuchsin Solution
5. Aniline Blue Solution
6. Bouin’s Solution
7. Weigert’s Iron Hematoxylin Solution Kit:
  - Stock Solution A
  - Stock Solution B
8. 1% Acetic Acid Solution in distilled water

### 2.4. Other Materials

1. Ethanol: 100%, 85%, 70%, and 50% (*v*/*v*) in distilled water.
2. Xylene
3. Acetone, cold (stored at −20 °C
4. Hydrophobic pen
5. Distilled water
6. Slide holder
7. Glass coverslips
8. Glass Coplin jars
9. Mounting solution (ClearVue Mountant XYL, cat# 8312-4, ThermoFisher Scientific, Waltham, MA, USA)
10. Rubber tube for connecting the faucet to allow continuous rinsing
11. Heart matrix
12. Razor blades

## 3. Procedure

### 3.1. Tissue Embedding

1. Euthanize the animals using CO_2_ per animal protocol guidelines.
2. Dissect out the hearts and thoroughly wash in PBS to remove all blood (3 times).
3. Cut the tissue into 2 mm thick cross-section or longitudinal sections using a heart slicer matrix and razor blades.

#### 3.1.1. Paraffin-Embedding and Sectioning

1. Place one slice into each tissue processing cassette.
2. Place the cassettes in 4% PFA solution for 1 h.
3. Place the cassettes in a beaker containing distilled water for 5 min.
4. Transfer the cassettes through the following solutions in sequence as shown in Table 1.
5. Gently with forceps, remove the tissue section from the cassette and transfer it to a sectioning mold. Pour molten paraffin to overfill the mold slightly. Lightly press down on the section to ensure it is flat in the mold.
6. Remove any air bubbles using forceps.
7. Allow the paraffin to solidify at room temperature on a level surface.
8. Prior to sectioning, place the paraffin block on ice for 2–3 h with the tissue surface facing the ice (bottom of the mold).
9. Mount the paraffin block in the microtome and cut sections of the desired thickness.
10. Using a pair of forceps, transfer the sections onto a 37 °C water bath.
11. Allow the sections to stretch completely and pick them up using a glass slide.
12. Place the slides for drying on a slide warmer at 37 °C overnight.
13. Store slides at room temp until ready to stain.

#### 3.1.2. Cryo-Embedding and Sectioning

1. Place a small amount of OCT compound in the tissue embedding mold.
2. Place one slice atop the OCT compound in the mold and lightly press down to ensure the section is flat.
3. Fill the mold entirely with OCT compound. Remove any air bubbles using a needle.
4. Incubate the sections in the OCT compound at room temperature for 15 min.
5. Snap-freeze the OCT compound by placing the molds on dry ice.
6. Store at −80 °C until sectioned.
7. For sectioning, mount the OCT block onto the cryostat and cut sections of the desired thickness.
8. Transfer the sections onto pre-chilled glass slides.
9. Store the slides at −80 °C until use.

### 3.2. Slide Preparation: Deparaffinization

Deparaffinize paraffin-embedded slides before staining. Use Coplin jars or an equivalent for each solution. Dip the slides in the following solutions sequentially, shown in Table 2.

Place slides in distilled water to wash off ethanol. Keep the slides in distilled water until ready for fixation and staining (see Appendix A, List item 1).

### 3.3. Fixation of Slides (Paraffin and Cryogenic)

For both paraffin-embedded and cryogenic slides, prior to staining, fix the slide by placing them in Bouin’s solution for 6 h at room temperature or 2 h at 56 °C. Do not exceed 6 h [see Appendix A, List item 2].

### 3.4. Staining with MTC

The protocol for staining cryogenic slides and paraffin-embedded slides is similar with a few minor differences.

**Figure 1:**
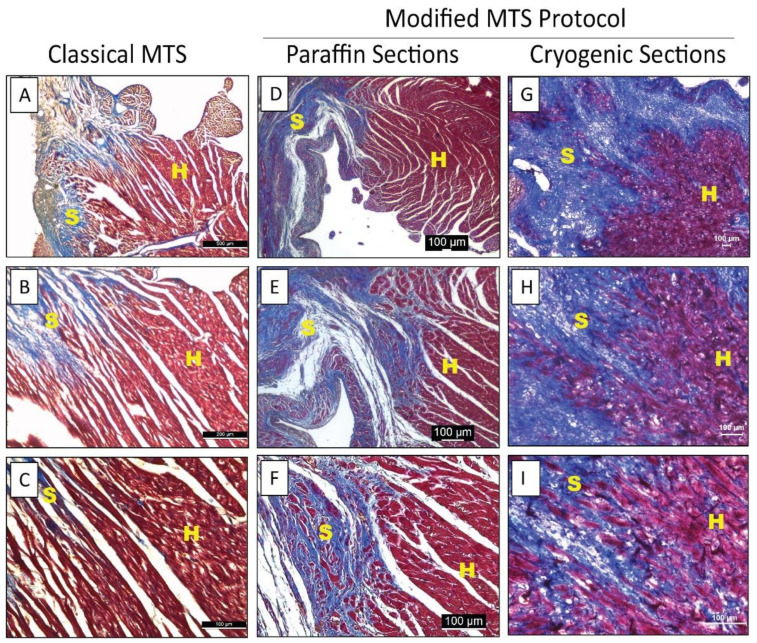
MTS images of heart sections. (**A**–**C**) Brightfield images showing fibrotic regions in post-MI heart in cryosections using classical MTS. (**D**–**F**) Brightfield images showing fibrotic region in post-MI heart in paraffin-embedded sections using the modified MTS protocol. (**G**–**I**) Brightfield images showing fibrotic regions in post-MI heart in cryosections using modified MTS. H indicates healthy tissue and S indicates fibrotic scar.

1. Rinse fixed slides with running tap water for 10 min to completely wash off the Bouin’s solution [see Appendix A, List items 3 and 4].
2. Place paraffin-embedded slides in cold acetone (−20 °C) for 3 min. Skip this step for cryogenic slides.
3. Mark the tissue sections with a hydrophobic pen to minimize reagent volumes to be used.
4. Make a working solution of Weigert’s Iron Hematoxylin by mixing equal volumes of Solution A and Solution B [see Appendix A, List item 5].
5. Add a working solution of Weigert’s Iron hematoxylin directly onto the tissue sections and incubate for 10 min [see Appendix A, List item 6].
6. Gently rinse the slides under running tap water for 5 min [see Appendix A, List item 4].
7. Add Biebrich scarlet-acid fuchsin solution to the slides for 10 min [see Appendix A, List items 6 and 7].
8. Wash off the stain by dipping the slides 3–5 times in distilled water for 2–3 s.
9. Make a phosphomolybdic-phosphotungstic acid solution by mixing phosphomolybdic acid solution, phosphotungstic acid solution, and distilled water at a ratio of 1:1:2, respectively [see Appendix A, List item 5].
10. Incubate the slides in the phosphomolybdic-phosphotungstic acid solution for 15 min [see Appendix A, List item 8].
11. Remove excess solution by gently tapping the slide on a paper towel. Do not rinse with distilled water.
12. Add aniline blue solution on the slide and incubate for 5 min.
13. Repeat step 8.
14. Incubate the slide in 1% acetic acid solution for 1 min.
15. Repeat step 8.
16. Dip the slides in 85% ethanol, 100% ethanol, and xylene for 5 s each [see Appendix A, List item 9].
17. Add a few drops of ClearVue Mountant Xyl (non-aqueous, xylene-based) mounting medium on each slide [see Appendix A, List item 10].
18. Place coverslip gently, ensuring no air bubbles are formed.
19. Store slides at room temperature.
20. Image the slides under a bright-field microscope (See Figure 1).

## 4. Expected Results

Proper MTS staining will provide a unique staining pattern to identify fibrosis in the tissue section. The heathy muscle tissue (H) can be identified by the bright red staining while the fibrotic scar region (S) can be identified by the blue staining (Figure 1).

## Figures and Tables

**Table 1:** The Sequential procedure to be followed for paraffin-embedding and sectioning of tissues.

Sequence	Procedure	Time (Minutes)
(i)	10% ethanol	2 × 15 min
(ii)	30% ethanol	2 × 15 min
(iii)	50% ethanol	2 × 15 min
(iv)	70% ethanol	2 × 15 min
(v)	85% ethanol	2 × 15 m
(vi)	100% ethanol	2 × 15 min
(vii)	Ethanol:xylene (1:1)	2 × 20 min
(viii)	Xylene	2 × 20 min
(ix)	Xylene:paraffin (1:1)	1 × 60 min (60 °C)
(x)	Paraffin	2 × 60 min (60 °C)

**Table 2:** Sequential steps to be followed for deparaffinization of slides.

Sequence	Procedure	Time (Min)
(i)	Xylene	2 × 3 min
(ii)	Ethanol:Xylene (1:1)	3 min
(iii)	100% Ethanol	3 min
(iv)	85% Ethanol	3 min
(v)	70% Ethanol	3 min
(vi)	50% Ethanol	3 min

**Figure A1:**
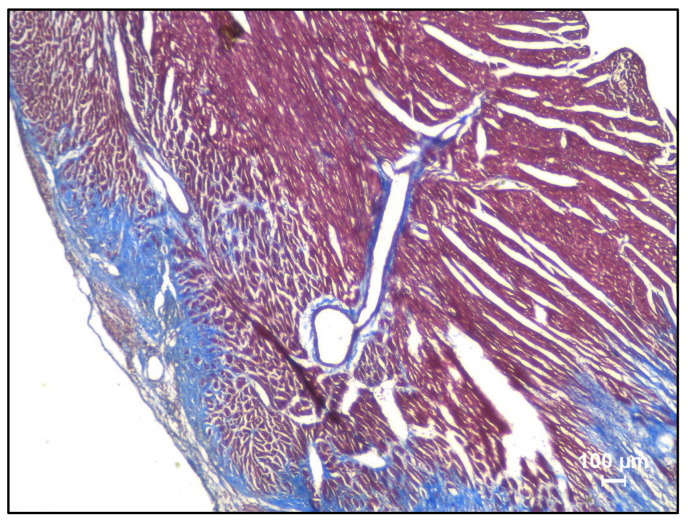
Representative image showing poor MTS due to shorter incubation times.

**Figure A2:**
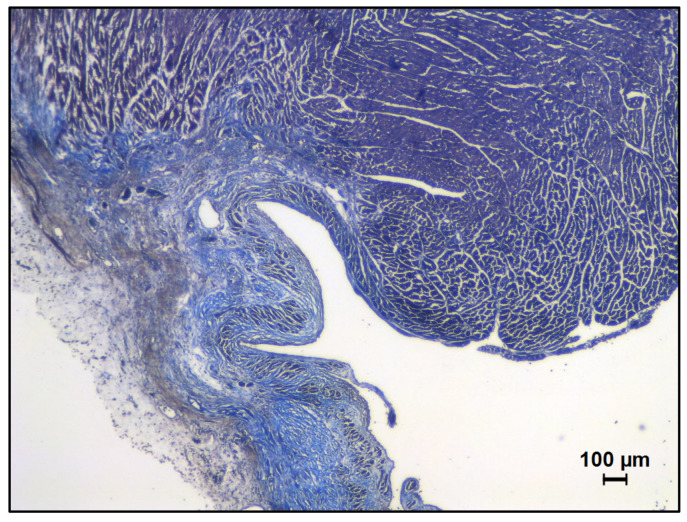
Representative image showing poor contrast in MTS of heart sections due to extended incubation in aniline blue.

## Data Availability

Not applicable.

## Appendix A

1. Inconsistent deparaffinization may result in improper staining. Ensure that solutions for deparaffinization are fresh and the slides are uniformly deparaffinized.
2. Incubating with Bouin’s solution can be tricky. If heated, it must be maintained at 56 °C. If kept at room temperature, incubation times should not exceed 6 h. Longer incubation times leave a yellowish tinge on the samples.
3. While rinsing the slides under running tap water, water pressure must be regulated. In addition, ensure that the tap water is not directly onto the sections. Excess pressure or water added directly to the sample can cause the sample to detach from the slide. Nevertheless, it is essential to ensure that the slide is fully immersed in water to wash off the stain completely. If a remnant stain is observed on the slide, increase the washing time.
4. In some instances, if a very faint tinge is observed after incubating the slides in Bouin’s Solution or Weigert’s Solution, the staining process can be continued further.
5. While staining solutions can be prepared in advance, making fresh staining solutions each time for best results is recommended.
6. At least 200 μL of stain must be used for each section to ensure complete coverage of the section. Add staining solutions dropwise onto the sections to ensure uniform coverage and consistent staining.
7. Depending on the sample type, longer incubation times may be required for deeper red color (Figure A1).
8. Staining in the phosphomolybdic-phosphotungstic acid solution for longer durations can wash off the scarlet-acid fuchsin. This step must be standardized for each tissue type (Figure A2).
9. Excess hydrophobic pen residue can be carefully wiped off after dipping.
10. The aqueous mounting medium must not be used for MTS samples.
